# Sulbactam-durlobactam improves cephalosporin and carbapenem susceptibility and time-kill effect against *Mycobacterium abscessus*

**DOI:** 10.1128/spectrum.01492-25

**Published:** 2025-09-10

**Authors:** Avneesh Shrivastava, Gunavanthi D. Boorgula, Sanjay Singh, Danaleigh Stiles, Pamela J. McShane, Megan Devine, Tawanda Gumbo, Shashikant Srivastava

**Affiliations:** 1 Division of Infectious Diseases, Department of Medicine, University of Texas at Tyler School of Medicine 675071, Tyler, Texas, USA; 2 Undergraduate Medical Education, University of Texas at Tyler School of Medicine 675071, Tyler, Texas, USA; 3 Section of Pulmonary and Critical Care, Department of Medicine, University of Texas at Tyler School of Medicine 675071, Tyler, Texas, USA; 4 IMPI Biotechnology Company Incorporated, Mount Hampden, Zimbabwe; 5 Department of Cellular and Molecular Biology, The University of Texas Health Science Centre at Tyler 12341, Tyler, Texas, USA; Innovations Therapeutiques et Resistances, Toulouse, France

**Keywords:** cephalosporins, carbapenems, MIC, nontuberculous mycobacteria

## Abstract

Despite the long therapy duration, the treatment outcomes for *Mycobacterium abscessus* lung disease (MAB-LD) are very poor. β-Lactams are among the recommended drugs for the treatment of MAB-LD; however, they are prone to hydrolysis by MAB β-lactamase enzymes. Therefore, β-lactamase inhibitors have been developed to overcome this problem. Here, we performed minimum inhibitory concentration studies with a reference MAB strain (ATCC19977) and a collection of 63 clinical isolates for penicillin, cephalosporin, and carbapenems alone or in combination with β-lactamase inhibitors, sulbactam/durlobactam (Sul/Dur). There was an average 64-fold (range: 19 to 103) reduction in drugs MIC in the presence of 4 mg/L Sul/Dur (1:1 ratio). The fold reduction in MICs was as follows: amoxicillin 94, ceftriaxone 103, cefuroxime 69, cefdinir 19, cefalexin 74, imipenem 23, meropenem 37, tebipenem 89, and faropenem 74. The Sul/Dur combination also improved the kill effect of the tested β-lactams by multiple folds as follows: amoxicillin 12.6, ceftriaxone 2,872, cefuroxime 135.5, cefdinir 26.5, cefalexin 1,030, imipenem and meropenem 14, and faropenem 5.5. Since imipenem is already included in the MAB-LD treatment recommendations and ceftriaxone achieves very high lung concentration, we propose to first test these drugs with Sul/Dur as a double β-lactam-β-lactamase backbone regimen to advance the therapy for MAB-LD.

**IMPORTANCE:**

β-Lactams are among the recommended drugs for the treatment of *Mycobacterium abscessus* lung disease (MAB-LD); however, they are prone to hydrolysis by MAB β-lactamase enzymes. We show that sulbactam/durlobactam (Sul/Dur) can improve the kill effect of several β-lactam antibiotics that, otherwise, due to observed high minimum inhibitory concentration, deem ineffective. Based on our findings, we propose imipenem that is already included in the MAB-LD treatment recommendations and ceftriaxone that achieves very high lung concentration, to test with Sul/Dur as a double β-lactam-β-lactamase backbone regimen to advance the therapy for MAB-LD.

## INTRODUCTION

Treatment of *Mycobacterium abscessus* (MAB), an environmental nontuberculous mycobacteria (NTM), lung disease (LD) is challenging due to intrinsic resistance to many antibiotics, including the recommended drugs in the multi-society treatment guidelines (1). Individuals with immunosuppression, underlying lung disease, and cystic fibrosis are at a higher risk of NTM infections, including MAB, and co-infection with other mycobacteria, and disease recurrence is very frequent (2). Despite the long therapy duration, the treatment outcomes for MAB-LD are poor, ranging between 25% and 58% (3–5). One meta-analysis of therapy duration and outcomes (6) reported the sputum culture conversion with macrolide-containing regimens as 34% among new MAB patients, and the estimated recurrence rate per month was 1.84% (95% confidence interval [CI]: 1.67% to 3.19%) (7).

β-Lactams are among the recommended drugs for the treatment of MAB-LD (1). However, the Bla_Mab_, a class A β-lactamase enzyme of MAB, hydrolyzes penicillins and cephalosporins, whereas carbapenems are comparatively less prone to hydrolysis (2). Several β-lactamase inhibitors have been developed to overcome this problem (2, 8–13). Here, we performed studies with sulbactam-durlobactam (herein referred to as “Sul/Dur”), commercially available as XACDURO (14). Sulbactam is a β-lactamase inhibitor, also considered a β-lactam, that works by inhibiting cell wall biosynthesis by blocking penicillin-binding proteins (PBPs), leading to cell death (14). Durlobactam is a novel broad-spectrum diazabicyclooctane non-β-lactam β-lactamase inhibitor that protects sulbactam from degradation by serine-β-lactamases (14). Since durlobactam also binds to PBPs, it is believed to have an antimicrobial effect of its own in addition to acting as a β-lactamase inhibitor.

The Sul/Dur combination is approved by the USA Food and Drug Administration for treatment of carbapenem-resistant *Acinetobacter baumannii* (CRAB); given as 1 g sulbactam and 1 g durlobactam every 6 h by intravenous (IV) infusion over 3 h in adults with a creatinine clearance (CLcr) of 45 to 129 mL/min, with ~2 h half-life for both (14). The pharmacokinetic/pharmacodynamic (PK/PD) parameter for sulbactam is % time above MIC (%T_/MIC_), and for durlobactam is 24-h unbound (*f*) AUC. The epithelial lining fluid (ELF) to plasma AUC ratio for sulbactam and durlobactam is 0.5 and 0.37, respectively (14).

There have been reports of β-lactams and novel β-lactamases combination against MAB (9, 10, 13, 15). In the present study, we tested penicillin (amoxicillin), cephalosporins (cefdinir, ceftriaxone, cefalexin, and cefuroxime), and carbapenems (imipenem, meropenem, tebipenem, and faropenem) with and without combination of Sul/Dur to determine its effect on the efficacy (E_max_ or maximum bacterial kill) and potency (EC_50_ or drug concentration required for 50% of the E_max_), and the change in the minimum inhibitory concentration (MIC) among MAB clinical isolates that would potentially increase the %T_/MIC_ if the MICs are lowered in combination of Sul/Dur.

## MATERIALS AND METHODS

### Bacteria, drugs, and other supplies

We used one reference strain of MAB (ATCC#19977) and a library of 63 species-identified clinical isolates. Middlebrook 7H9 broth supplemented with 10% oleic acid-albumin-catalase–dextrose (OADC) (herein termed “7H9 broth”) and cation-adjusted Mueller Hinton broth (herein termed “CAMHB”) were used in the experiments (16). Drugs were purchased from the UT Health Science Center at Tyler campus pharmacy and BOC Sciences (NY, USA). The drug was reconstituted in 0.9% normal saline to get the desired concentrations used in the respective experiments.

### Minimum inhibitory concentration determination

First, we performed broth micro-dilution MIC experiments (16), only with the ATCC19977 strain, in both 7H9 and CAMHB to determine any differences in the MICs based on the growth medium used in the experiments. MICs of Sul/Dur were determined as a single drug and two-drug combination (1:1 ratio). The concentration ranged from 0.06 to 256 mg/L in a two-fold dilution. Second, MICs of amoxicillin, cephalosporins (ceftriaxone, cefdinir, cefalexin, cefuroxime), and carbapenems (imipenem, meropenem, faropenem, and tebipenem) against 63 clinical isolates were performed with and without a combination of 4 mg/L sulbactam-durlobactam.

Briefly, logarithmic phase growth cultures were used to prepare the inoculum by adjusting the turbidity to McF Standard 0.5 using a densitometer, followed by back-dilution in the culture medium to get a starting bacterial burden of ~10^5^ CFU/mL. Next, we added 198 µL of the inoculum to each well of 96-well sterile, round-bottom plates pre-filled with 2 µL (100×) of each drug with concentrations as mentioned above. Plates were sealed in a Ziplock bag to prevent evaporation during the 72-h incubation at 30°C. At the end of the incubation period, visual MICs were recorded using an inverted mirror. The MICs were read by three individuals. The lowest drug concentration inhibiting visible bacterial growth was recorded as the MIC. In instances where a trailing effect was observed, a consensus between two readers was needed to record the MIC values. Each MIC experiment was performed twice, with two replicates for each concentration.

### Static concentration-response studies

In the abbreviated time-kill curve, two time points are considered: t = 0 or the inoculum, and at the end of co-incubation at 72 h, without addressing day one or two bacterial burden. We performed static concentration-response studies with sulbactam-durlobactam and each of the study drugs alone or in combination to determine the extent of change in the drug’s maximum effect (E_max_), and concentration that achieves 50% of E_max_ (EC_50_) when combined with 4 mg/L Sul/Dur. The inoculum preparation was as described for the MIC experiments, so was the drug concentration range. There were two replicates per drug concentration. Experiments were set in a total volume of 5 mL, where 50 µL (100×) of each concentration was added to the 4,950 µL of the inoculum. Cultures were incubated for 72 h at 30°C with gentle shaking. Next, 1 mL of the cultures was collected in a sterile centrifuge tube, carry-over drug was removed by washing twice with normal saline by centrifuging at 13,000 rpm for 5 min. Bacterial pellets were resuspended in 1 mL saline to prepare 10-fold serial dilutions, and samples were inoculated on 7H10 agar for CFU estimation. Agar plates were sealed in a Ziplock, and CFUs were recorded after 72 h of incubation at 30°C.

### Data analysis

MS-EXCEL was used to record the MIC of the reference strain and 63 clinical isolates. The MIC distribution was used to calculate the cumulative MICs to identify the MIC_50_ and MIC_90_ of each drug alone or in combination with sulbactam-durlobactam. GraphPad Prism version 10.4.2 was used for the inhibitory sigmoid E_max_ model between the drug concentrations and the bacterial burden.

## RESULTS

### MIC of β-lactams with and without Sul/Dur

**Fig 1:**
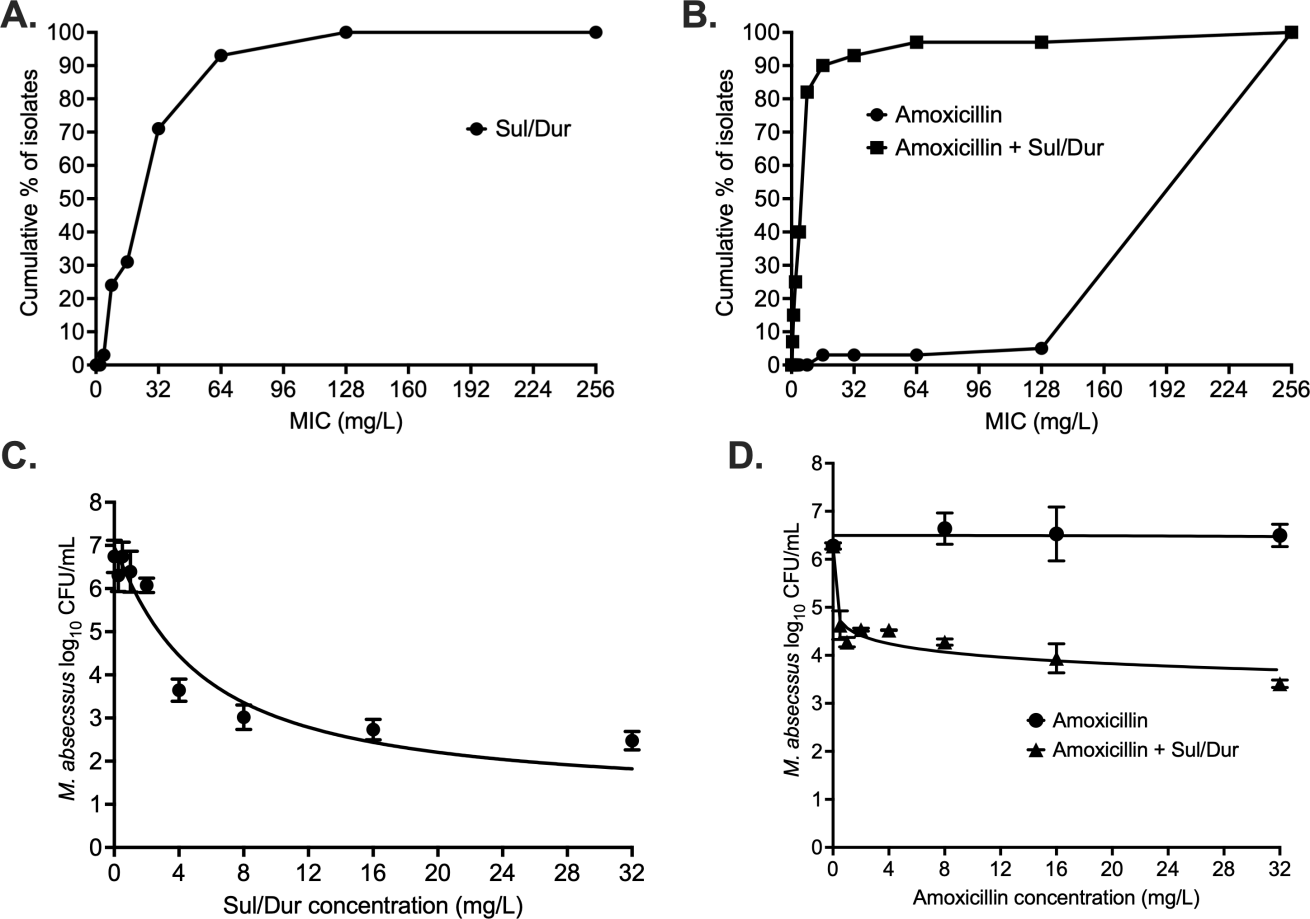
Concentration-response and MIC distribution. (**A**) Sulbactam-durlobactam MIC distribution among clinical isolates. (**B**) Amoxicillin MIC distribution with and without 4 mg/L sulbactam-durlobactam. (**C**) Sulbactam-durlobactam model fit. (**D**) Comparison of model fit curves for amoxicillin alone and in combination with sulbactam-durlobactam. (Sul/Dur, sulbactam-durlobactam).

For the reference ATCC strain using the CAMHB, the MIC of sulbactam was >128 mg/L, durlobactam MIC was 64 mg/L, and MIC for the Sul/Dur combination was 64 mg/L. In comparison, the sulbactam MIC in 7H9 broth remained >128 mg/L, whereas durlobactam MIC changed to 8 mg/L, and MIC for the Sul/Dur combination was recorded as 4 mg/L. Therefore, subsequent MIC experiments with clinical isolates were performed using the 7H9 broth. The Sul/Dur MIC distribution among the clinical isolates is shown in Fig. 1A. Table S1 lists the MICs for individual isolates ranging from 4 to 128 mg/L. MIC_50_ and MIC_90_ for the Sul/Dur combination were calculated as 32 and 64 mg/L, respectively.

Amoxicillin alone MICs among the clinical isolates ranged between 128 to 256 mg/L, and MIC_50_ and MIC_90_ were calculated as 256 mg/L. However, when amoxicillin was tested in combination with Sul/Dur, the MICs ranged between 0.5 and 256 mg/L, and MIC_50_ and MIC_90_ as 8 and 16 mg/L, respectively. The amoxicillin MICs for individual isolates are listed in Table S1, whereas MIC distribution results are summarized in Fig. 1B.

**Fig 2:**
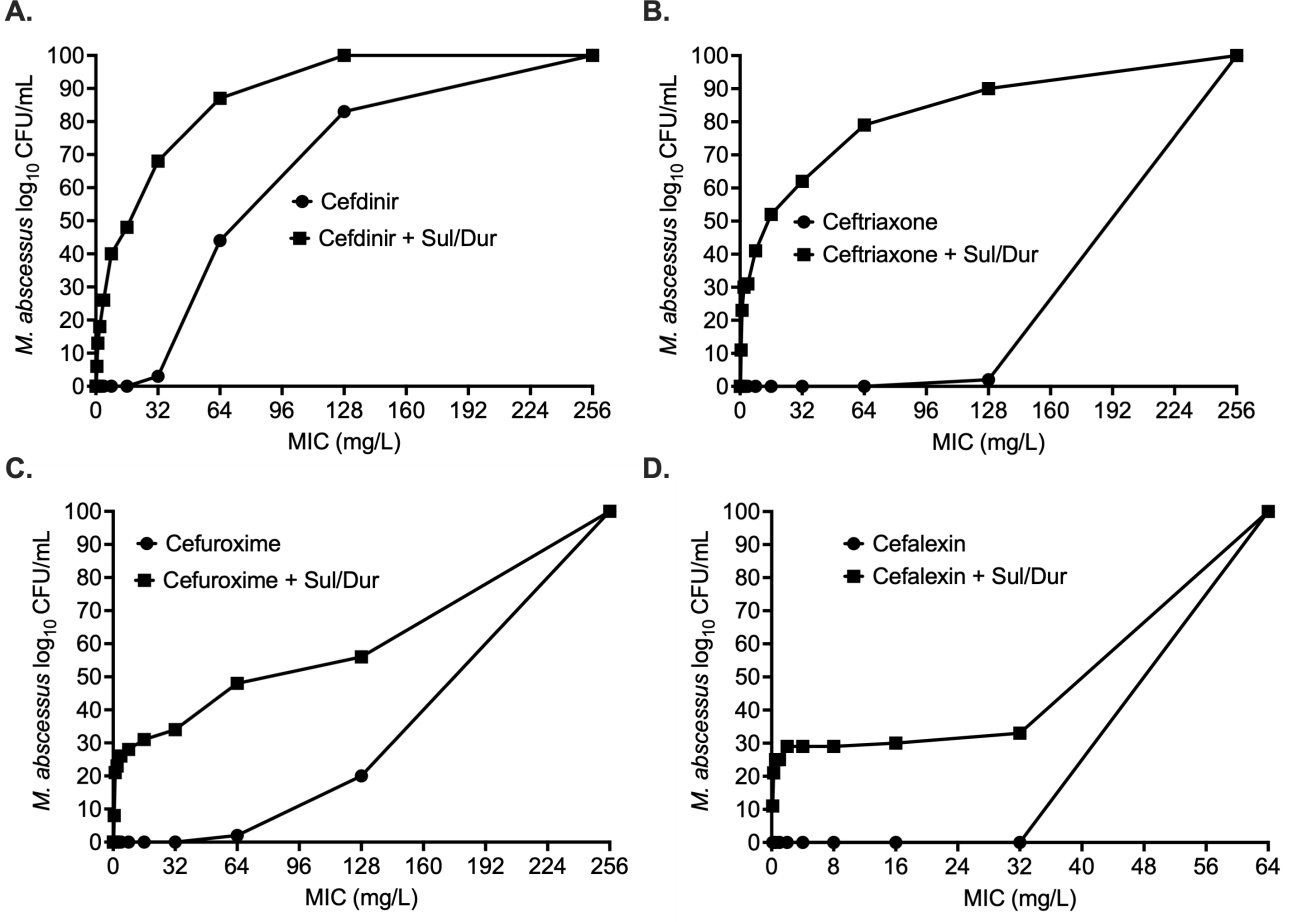
Cephalosporin MIC distribution. Sulbactam-durlobactam, combined at a fixed concentration (1:1 ratio) of 4 mg/L resulted in lowering the MIC with each of the four cephalosporins tested against 63 clinical isolates: (**A**) cefdinir, (**B**) ceftriaxone, (**C**) cefalexin, and (**D**) cefuroxime. MIC distribution of each drug for each individual isolated is listed in Table S1 (Sul/Dur, sulbactam-durlobactam).

Among the cephalosporins, cefdinir MIC distribution is shown in Fig. 2A, highlighting a four- and two-fold reduction in MIC_50_ and MIC_90_, respectively, in the presence of Sul/Dur. Table S1 lists individual isolate cefdinir MICs with and without Sul/Dur. Ceftriaxone MIC distributions are shown in Fig. 2B and Table S1, displaying a 16- and 2-fold reduction in MIC_50_ and MIC_90_, respectively, when combined with Sul/Dur. For cefuroxime, the MIC distribution is shown in Fig. 2C and Table S1. The cefuroxime MICs of all the clinical isolates were 64 mg/L; however, in combination with Sul/Dur, they ranged from 0.125 to 64 mg/L. Cefalexin MIC distribution is shown in Fig. 2D and Table S1. The MIC_50_ and MIC_90_ were 256 mg/L for cefalexin alone. However, we observed a two-fold reduction in the cefalexin MIC_50_ when combined with Sul/Dur, but the cefalexin MIC_90_ remained unchanged.

**Fig 3:**
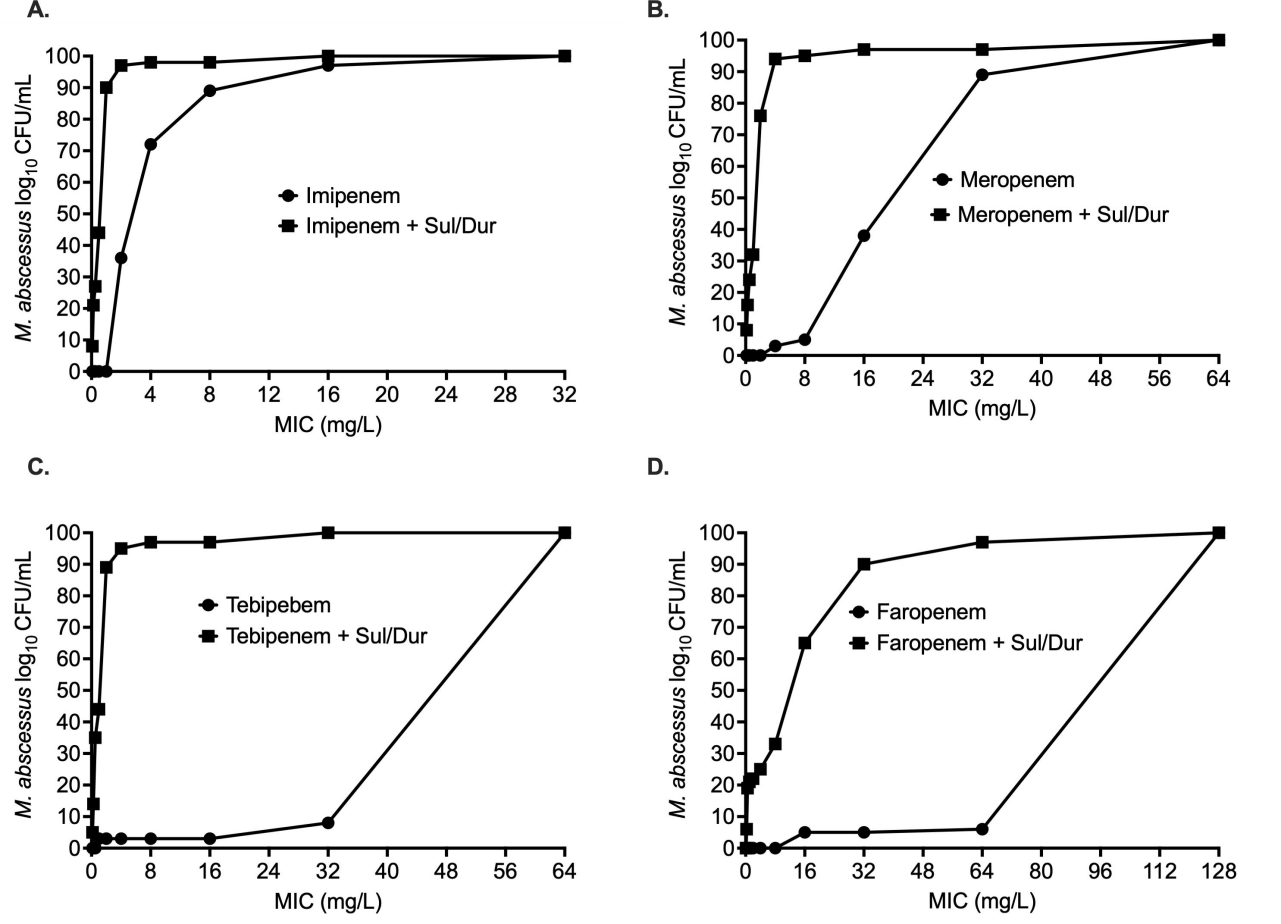
Carbapenem MIC distribution. Sulbactam-durlobactam, combined at a fixed concentration (1:1 ratio) of 4 mg/L, resulted in lowering the MIC with each of the four carbapenems tested against 63 clinical isolates: (**A**) imipenem, (**B**) meropenem, (**C**) tebipenem, and (**D**) faropenem. MIC distribution of each drug for each individual isolated is listed in Table S1 (Sul/Dur, sulbactam-durlobactam).

Regarding the carbapenems, imipenem MIC distribution is shown in Fig. 3A with an 8- and 16-fold reduction in MIC_50_ and MIC_90_, respectively, in the presence of Sul/Dur. Meropenem MIC_50_ and MIC_90_ were 32 and 64 mg/L, respectively, that changed to 2 and 4 mg/L, respectively, in the presence of 4 mg/L Sul/Dur (Fig. 3B). We observed a multiple-fold reduction in MIC_50_ and MIC_90_ of tebipenem in the presence of Sul/Dur (Fig. 3C). Sul/Dur combination also improved faropenem MICs, where MIC_50_ and MIC_90_ were recorded as 16 and 32 mg/L, respectively (Fig. 3D).

Table S1 summarizes the fold change in MIC individual isolates for each cephalosporin and carbapenem when combined with Sul/Dur. Overall, there was a mean 64-fold change in β-lactams MICs in the presence of Sul/Dur. The rank order of the drugs by fold change in the MICs was ceftriaxone (103-fold) > amoxicillin (94-fold) > tebipenem (89-fold) > cefuroxime and faropenem (both 74-fold) > cefalexin (59-fold) > meropenem (37-fold) > cefdinir (19-fold).

### Static concentration-response studies with and without Sul/Dur

The results of the Sul/Dur (1:1 ratio) concentration-response study are shown in Fig. 1C, with inhibitory sigmoid maximal effect model parameters summarized in Table 1. The E_max_ with Sul/Dur was 5.84 ± 0.51 log_10_ CFU/mL, and EC_50_ calculated as 5.17 ± 1.02 mg/L.

**TABLE 1:** Inhibitory sigmoid maximal effect model parameters for sulbactam-durlobactam and other study drugs^*a*^

Drug	E_con_	E_max_	H	EC_50_	r^2^
Sul/Dur	6.96 ± 0.27	5.84 ± 0.51	1.09 ± 0.22	5.17 ± 1.02	0.92
Penicillin					
Amoxicillinn	6.49 ± 0.09	2.28 ± 0.68	2.25 ± 1.09	243.9 ± 83.13	0.90
Amoxycillin + Sul/Dur	6.26 ± 0.25	2.28 ± 0.28	0.18 ± 0.36	239.5 ± 9737	0.91
Cephalosporins					
Cefdinir	6.93 ± 0.23	3.58 ± 0.30	2.42 ± 0.65	25.15 ± 3.31	0.095
Cefdinir + Sul/Dur	6.52 ± 0.17	4.41 ± 1.87	0.37 ± 0.27	0.95 ± 2.24	0.92
Ceftriaxone	6.47 ± 0.14	3.83 ± 1.51	1 (fixed)	430.8 ± 337.8	0.86
Ceftriaxone + Sul/Dur	6.18 ± 0.29	2.35 ± 0.33	1 (fixed)	0.15 ± 0.11	0.80
Cefalexin	7.28 ± 0.13	0.88 ± 0.15	1 (Fixed)	2.06 ± 1.26	0.72
Cefalexin + Sul/Dur	7.32 ± 0.23	2.84 ± 0.26	1 (Fixed)	0.002 ± 0.05	0.91
Cefuroxime	7.18 ± 0.09	3.51 ± 0.13	3.49 ± 0.50	46.08 ± 2.58	0.99
Cefuroxime + Sul/Dur	7.20 ± 0.14	3.68 ± 0.22	0.84 ± 0.24	0.34 ± 0.07	0.98
Carbapenems					
Imipenem	7.01 ± 0.22	4.35 ± 0.26	1.87 ± 0.34	0.85 ± 0.34	0.97
Imipenem + Sul/Dur	6.96 ± 0.24	5.36 ± 0.24	0.17 ± 0.62	0.06 ± 1.16	0.94
Meropenem	7.01 ± 0.22	4.35 ± 0.26	1.87 ± 0.34	1.69 ± 0.18	0.97
Meropenem + Sul/Dur	6.96 ± 0.24	5.36 ± 0.24	0.17 ± 0.62	0.12 ± 2.32	0.94
Faropenem	6.84 ± 0.58	2.34 ± 0.23	1.35 ± 1.28	116.0 ± 515.7	0.86
Faropenem + Sul/Dur	6.84 ± 0.58	3.92 ± 0.27	0.19 ± 0.44	21.01 ± 679	0.89
Tebipenem	6.68 ± 0.00	6.68 ± 0.00	0.29 ± 0.42	10827 ± 519851	0.79
Tebipenem + Sul/Dur	6.72 ± 0.18	4.79 ± 0.68	0.52 ± 0.19	0.58 ± 0.29	0.97

^*a*^ Sulbactam-durlobactam, Sul/Dur; Units for E_con_ and E_max_ are log_10_ CFU/mL; EC_50_ in mg/L.

In the static concentration-response studies, we tested nine β-lactams in combination with 4 mg/L Sul/Dur. The MAB kill below stasis (inoculum or day 0 bacterial burden) with 4 mg/L Sul/Dur was 0.52 ± 0.44 log_10_ CFU/mL. Fig. 1D shows the inhibitory sigmoid maximal effect model fitting curve for amoxicillin with parameters listed in Table 1. Amoxicillin alone did not kill MAB below stasis, but in combination with Sul/Dur, there was 0.62 ± 0.19 log_10_ CFU/mL MAB kill below stasis. The amoxicillin EC_50_ with and without Sul/Dur was calculated as 243.9 ± 83.12 and 238.5 ± 9737 mg/L, respectively.

**Fig 4:**
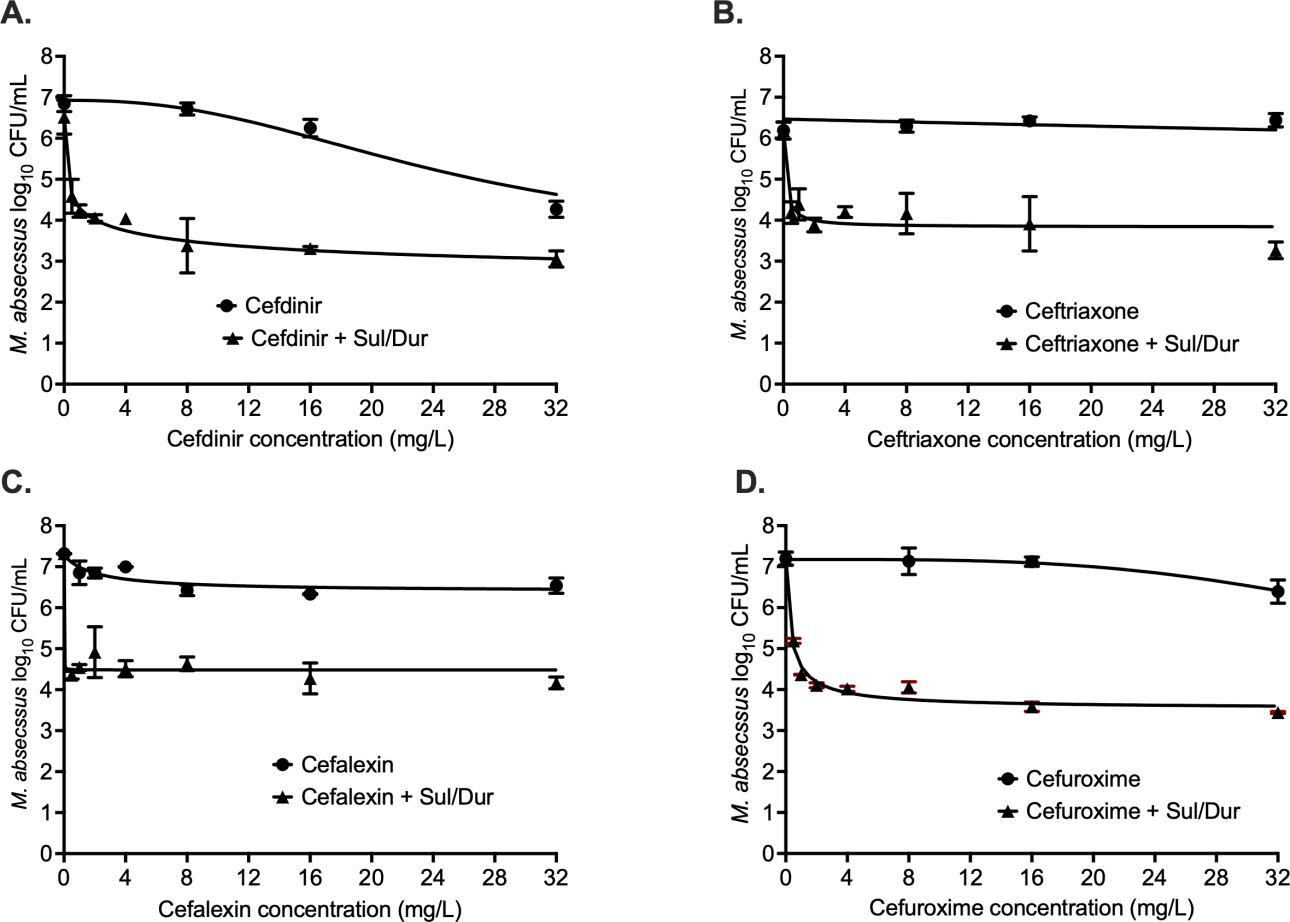
Cephalosporin concentration-response studies. Addition of sulbactam-durlobactam resulted in improved *M. abscessus* kill and lowered effective concentration (EC_50_) associated with maximal kill (E_max_) with each of the four cephalosporins. (**A**) Cefdinir killed 2.16 ± 46 log_10_ CFU/mL MAB below stasis, (**B**) ceftriaxone MAB kill below stasis was 1.66 ± 0.11 log_10_ CFU/mL, (**C**) cefalexin MAB kill below stasis was 0.97 ± 0.02 log_10_ CFU/mL, and (**D**) cefuroxime MAB kill below stasis was 1.75 ± 0.09 log_10_ CFU/mL. Detailed results for different model parameters are summarized in Table 1 (Sul/Dur, sulbactam-durlobactam).

Figure 4 and Table 1 show the results of the cephalosporin concentration-response studies. Cefdinir (Fig. 4A) E_max_ with and without Sul/Dur was not statistically different (3.58 ± 0.30 vs 4.41 ± 1.87 log_10_ CFU/mL); however, Sul/Dur lowered the cefdinir EC_50_ by >26.5-fold (Table 1). Figure 4B and Table 1 show the results for ceftriaxone, with E_max_ with and without Sul/Dur as 3.83 ± 0.14 and 2.35 ± 0.33 log_10_ CFU/mL, respectively. Ceftriaxone EC_50_ in combination with Sul/Dur lowered by an impressive 2,872-fold. Figure 4C shows the results for cefalexin, where the E_max_ with cefalexin alone was 0.88 ± 0.15 log_10_ CFU/mL, and Sul/Dur increased the E_max_ to 2.84 ± 0.26 log_10_ CFU/mL, a 3-fold change in kill effect (Table 1). The results for cefuroxime are shown in Fig. 2D, where E_max_ was not different with and without Sul/Dur (3.51 ± 0.13 vs 3.68 ± 0.22 log_10_ CFU/mL), but the EC_50_ improved by >135-fold (Table 1).

**Fig 5:**
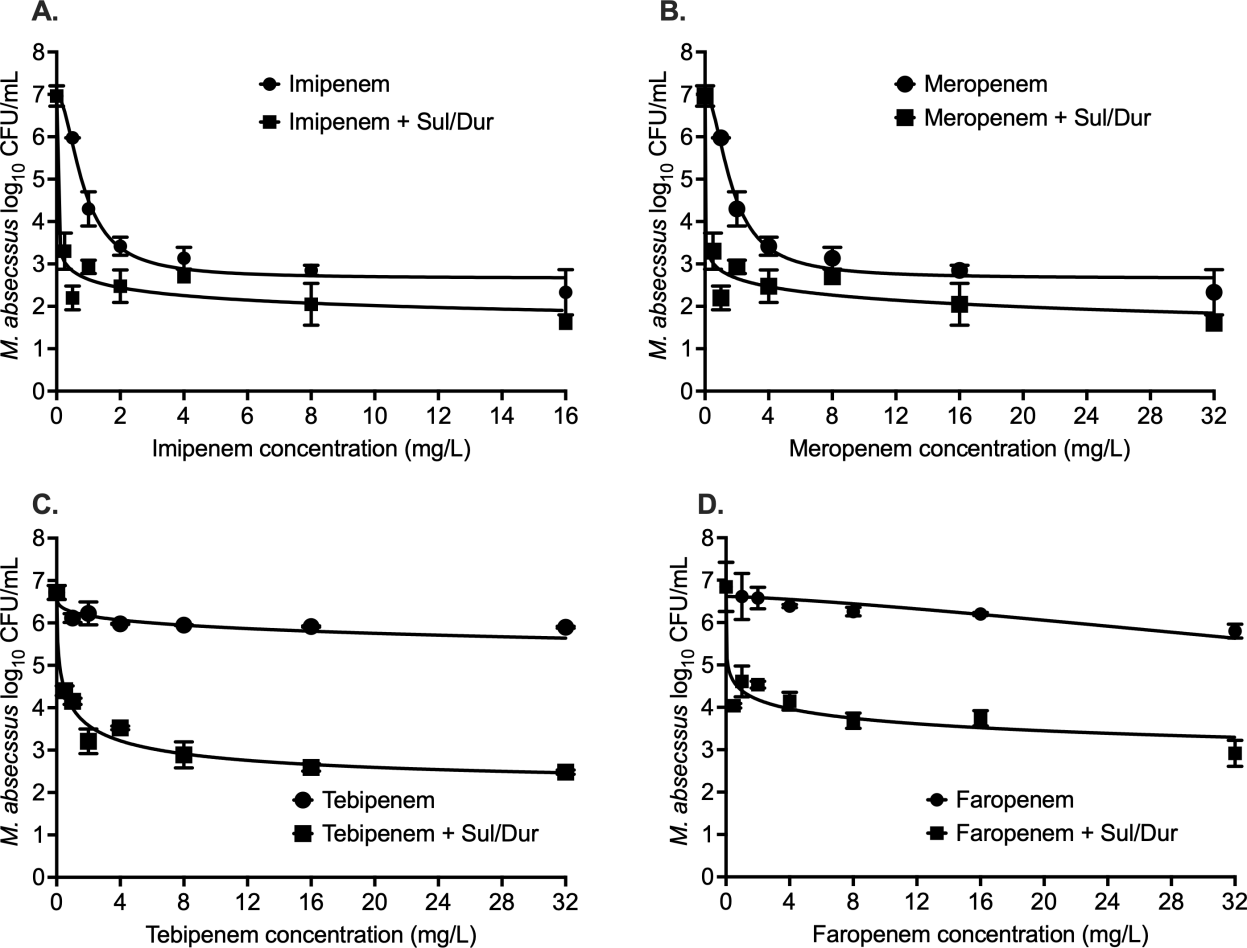
Carbapenem concentration-response studies. Addition of sulbactam-durlobactam resulted in improved *M. abscessus* kill and lowered effective concentration (EC_50_) associated with maximal kill (E_max_) with each of the four carbapenems. (**A**) Imipenem MAB kill below stasis was 4.34 ± 1.12 log_10_ CFU/mL, (**B**) meropenem MAB kill below stasis was 4.47 ± 1.35 log_10_ CFU/mL, (**C**) tebipenem MAB kill below stasis was 2.60 ± 0.01 log_10_ CFU/mL, and (**D**) faropenem MAB kill below stasis was 2.23 ± 0.38 log_10_ CFU/mL. Detailed results for different model parameters are summarized in Table 1 (Sul/Dur, sulbactam-durlobactam).

The results of the carbapenem concentration-response studies are summarized in Fig. 5, and Table 1 lists the inhibitory sigmoid model parameters. Figure 5A shows the results for imipenem, where E_max_ with and without Sul/Dur were not statistically different, so was the EC_50_ 0.84 ± 0.09 mg/L vs 0.06 ± 1.16 mg/L, considering standard deviations (Table 1). Figure 5B shows the results for meropenem concentration response, where Sul/Dur improved the meropenem EC_50_ by 14-fold (Table 1). The results for tebipenem are shown in Fig. 5C, where the model fit for tebipenem alone was poor. The E_max_ and EC_50_ with Sul/Dur were 4.78 ± 0.68 log_10_ CFU/mL and 0.58 ± 0.29 mg/L, respectively (Table 1).

All β-lactams killed MAB below stasis when combined with Sul/Dur. The rank order of the drugs by increase in the E_max_ (log_10_ CFU/mL) was #1 imipenem and meropenem (both 5.36 ± 0.24 log_10_ CFU/mL), #2 tebipenem (4.79 ± 0.68 log_10_ CFU/mL), #3 cefdinir (4.41 ± 1.87 log_10_ CFU/mL), #4 faropenem (3.92 ± 0.27 log_10_ CFU/mL), #5 cefuroxime (3.68 ± 0.22 log_10_ CFU/mL), #6 cefalexin (2.84 ± 0.26 log_10_ CFU/mL), #7 ceftriaxone (2.35 ± 0.33 log_10_ CFU/mL), and #8 amoxicillin (2.28 ± 0.28 log_10_ CFU/mL). However, the rank order of the drugs by change (lowering) in EC_50_ was different: ceftriaxone (2,872-fold) > cefalexin (1,030-fold) > cefuroxime (135.5-fold) > cefdinir (26.6-fold) > amoxicillin (94-fold) > imipenem and faropenem (both 14-fold) > faropenem (5.5-fold). Overall, these results suggest that β-lactams in combination with Sul/Dur warrant further exploration to develop new therapeutic regimens for the treatment of MAB lung disease.

## DISCUSSION

*Mycobacterium abscessus* subspecies are intrinsically resistant to many antibiotics, including β-lactams, and even when susceptible develop resistance quickly both *in vitro* and in patients. The rapid development of resistance is due to antibiotic-modifying enzymes, such as the β-lactamase, Bla_Mab_. Multiple reports suggest that by combining two β-lactams with or without a β-lactamase inhibitor, it is possible to hit multiple targets in cell wall synthesis pathway (12, 17, 18). There are some clinical reports describing the effectiveness and role of the dual β-lactam combinations (ceftaroline plus imipenem and imipenem-cilastatin plus amoxicillin) in the treatment of MAB-LD (19, 20). These findings suggest a target redundancy, that is, β-lactams and β-lactamase inhibitors occupy different penicillin-binding proteins and Bla_Mab_ with different affinities leading to inactivation of each target by two or more drugs (2, 12, 18, 21–24)

Sulbactam and durlobactam, as separate entities, as well as in combination with other β-lactams, have been shown to be effective against MAB in test tubes (11, 17, 25, 26). Elsewhere, Dousa et al. demonstrated that the MICs of amoxicillin, imipenem, and cefuroxime were dramatically reduced by durlobactam to MIC_50_ of ≤0.06 mg/L and MIC_90_ of 0.25 µg/mL (17). These authors also tested MICs in 54 MAB isolates and performed time-kill studies of the β-lactams sulopenem and cefuroxime, and combinations with the Bla_Mab_ inhibitors of durlobactam or avibactam (26). Sulopenem-cefuroxime combination lowered MIC_50_ and MIC_90_ to ≤0.25 µg/mL, while in time-kill studies, the sulopenem-cefuroxime combination killed ~1.5 log_10_ CFU/mL of MAB; addition of avibactam further reduced the bacterial burden: 3 log_10_ CFU/mL kill versus 4 log_10_ CFU/mL kill on addition of durlobactam (26). Indeed, these effects are across a large range of antibiotics, six in a study by Shin et al., and 15 in the study by Sayed et al., such that an exercise to find the best companion drugs for Sul/Dur is necessary, prior to exploring full PK/PD studies.(24, 27) In the present study, we report the effect of Sul/Dur on nine different β-lactams to advance our understanding and enrich the current literature on the synergistic effects of the dual β-lactams and potential role in the treatment of MAB-LD. In Table S2, we also show MICs of clarithromycin and azithromycin on a subset of 20 MAB clinical isolates to demonstrate that sul/dur can be used irrespective of macrolide resistance.

In the present study, first, we showed that the culture medium affects the Sul/Dur MICs. The sulbactam MIC of the ATCC strain in the CAMHB and 7H9 was >128 mg/L. However, the MIC of durlobactam was 64 mg/L in CAMHB and 8 mg/L in 7H9. The media effect was also observed when Sul/Dur was tested as a 1:1 combination: 64 mg/L in CAMHB vs 4 mg/L in 7H9. Therefore, it is important to determine the culture medium for any new drug as part of the drug susceptibility testing method developments (16). In the subsequent experiments with 7H9, the Sul/Dur MICs for the clinical isolates ranged from 4 to 128 mg/L, and the MIC_50_ and MIC_90_ were 32 and 64 mg/L, respectively.

Second, for amoxicillin (penicillin), the MIC_50_ and MIC_90_ were 256 mg/L, similar to that reported recently by Dousa et al. (17). The amoxicillin MIC_50_ and MIC_90_ were changed to 8 and 16 mg/L, respectively, in the presence of 4 mg/L sulbactam-durlobactam (1:1 ratio), which was higher compared to the MIC distribution reported using 8 mg/L durlobactam elsewhere (17). Thus, in agreement with the recent reports (13, 17), Sul/Dur indeed improved amoxicillin’s activity (MIC) against both the drug-susceptible reference strain and clinical isolates of MAB.

Third, we found that Sul/Dur consistently improved the potency (EC_50_) of cephalosporins; cefdinir EC_50_ lowered by >26-fold, ceftriaxone by 2,872-fold, cefuroxime by >135-fold, and cefalexin by 1,030-fold. Similarly, the change in cephalosporins MICs in the presence of Sul/Dur was remarkable: cefdinir 19-fold, ceftriaxone 103-fold, cefuroxime 69-fold, and cefalexin by 74-fold. These results are in agreement with previous *in vitro* studies reporting a reduction in cephalosporin’s MIC when tested in combination of sulbactam, durlobactam, or as a Sul/Dur combination (11–13, 26). Considering the change in the MIC (103-fold) and improved EC_50_ (2,872-fold) in combination with Sul/Dur, we propose to advance ceftriaxone to create a backbone regimen for MAB-LD, first testing in dynamic models, such as hollow fiber model of MAB (HFS-MAB), followed by clinical validation. Ceftriaxone is more β-lactamase stable with a longer half-life (6–8 h), has good lung to serum penetration ratios (~12×), and is also safe during pregnancy as well as children and infants out of the neonatal period (28–30).

Fourth, Sul/Dur was found to equally improve the performance of both old and new carbapenems, as has been reported by others (11, 13, 17). Imipenem is one of the recommended drugs in the guidelines for the treatment of MAB-LD, and we have shown elsewhere imipenem’s efficacy in the HFS-MAB and in clinics even when used in patients with refractory disease. Here, we found an average of 23-fold reduction in imipenem MICs and 14-fold lower EC_50_ when tested in combination of Sul/Dur. Similar improvements in meropenem MIC and EC_50_ in the presence of 4 mg/L Sul/Dur were observed as 37- and 14-fold, respectively. For tebipenem, an average of 89-fold reduction in MIC was noted; however, due to poor model fit, a comparison of EC_50_ could not be made, which was 0.58 + 0.29 mg/L in combination of Sul/Dur. Sul/Dur also improved both MIC and potency of faropenem.

In summary, Sul/Dur was found to affect the activity of β-lactams by reducing the MICs by multiple-fold, improving the MAB kill, and lowering the EC_50_ against MAB. Among the cephalosporins and carbapenems, considering the results presented here and previously published, we propose to advance ceftriaxone and imipenem to further test as double β-lactam-β-lactamase inhibitor backbones, followed by the addition of other active drugs to create multidrug combination regimens for effective treatment of MAB-LD.

## Data Availability

The raw data for the results presented in the article are available from the corresponding author, upon reasonable request following UTHSCT’s data-sharing policy.

## SUPPLEMENTAL MATERIAL

Supplemental tables spectrum.01492-25-s0001.xlsx Tables S1 and S2.

ASM does not own the copyrights to Supplemental Material that may be linked to, or accessed through, an article. The authors have granted ASM a non-exclusive, world-wide license to publish the Supplemental Material files. Please contact the corresponding author directly for reuse.
